# A randomized controlled trial of CBT therapy for adults with ADHD with and without medication

**DOI:** 10.1186/1471-244X-12-30

**Published:** 2012-04-05

**Authors:** Margaret Weiss, Candice Murray, Michael Wasdell, Brian Greenfield, Lauren Giles, Lily Hechtman

**Affiliations:** 1 University of British Columbia, Faculty of Medicine, 1488 Gordon Ave, West Vancouver, BC V7T 1R6, Canada; 2 Provincial ADHD Program, BC Mental Health and Addictions Services, Box 178, 4500 Oak St., Vancouver, BC V6H 3N1, Canada; 3 Bridepoint Health, 14 St. Matthews Road, Toronto, ON M4M 2B5, Canada; 4 McGill University Faculty of Medicine, Montreal Children's Hospital, 2300 Tupper St, Montreal, QC H3H 1P3, Canada; 5 University of British Columbia, Department of Psychiatry, 1488 Gordon Avenue, West Vancouver, BC V7T 1R6, Canada

**Keywords:** CBT, Adult, Attention-Deficit/Hyperactivity Disorder, Psychotherapy

## Abstract

**Background:**

Previous studies of psychological treatment in adults with ADHD have not controlled for medication status and include either medicated participants or mixed samples of medicated and unmedicated participants. The objective of this study is to examine whether use of medication improves outcome of therapy.

**Method:**

This was a secondary analysis comparing 23 participants randomized to CBT and Dextroamphetamine vs. 25 participants randomized to CBT and placebo. Both patients and investigators were blind to treatment assignment. Two co-primary outcomes were used: ADHD symptoms on the ADHD-RS-Inv completed by the investigator and improvement in functioning as reported by the patient on the Sheehan Disability Scale.

**Results:**

Both groups showed robust improvement in both symptoms and functioning, but the use of medication did not significantly improve outcome over and above use of CBT and placebo.

**Conclusion:**

This study replicates previous work demonstrating that CBT is an effective treatment for ADHD in adults. Within the limits of this pilot, secondary analysis we were not able to demonstrate that medication significantly augments the outcome of CBT therapy for adults with ADHD. The study was funded by GlaxoSmithKline, Clinical Trials Registry #GSK707.

## Background

Adult ADHD is a common and impairing condition [1]. Even though medications are very useful in treating symptoms of ADHD, there are a number of reasons why psychological treatment may also be required. Clinical trials of new medications for ADHD have demonstrated that up to one-quarter of patients will either not respond or not tolerate the medication, or if they do respond will improve but not achieve full remission [2,3]. Even when medication is effective, training in the acquisition of adaptive life skills may offer additional benefit, especially when looking at functioning in addition to symptom outcomes. ADHD throughout the life span is associated with self-perception of inadequacy and incompetence, and even if ADHD symptoms improve in adults, poor self esteem may remain embedded in the patient's self-concept [4].

Psychological treatments of ADHD in childhood in combination with medication shows a small margin of additional benefit over medication alone, especially in children with anxiety or other comorbid disorders [5]. There are however, several reasons to suspect that adults may be more responsive to psychological interventions than children. Children may be placed in therapy at the parents request, where adults are self-referred. By adulthood, many patients have obtained greater insight into their difficulties with ADHD and are receptive to learning better ways of coping. Children on medication are in school where they are exposed to demands and training in attention, where an adult on medication is unlikely to receive instruction in executive function or other skills.

Previous empirical research in psychological treatment in ADHD has consistently demonstrated that skills based therapies are effective for ADHD symptoms and functioning, despite differences in the details of the therapy offered. This is not true for insight oriented therapy which, in one study, had deleterious effects [6]. This is true for individual CBT [7-10] group psychoeducation and organization training [11], group cognitive remediation as used with brain injury patients [12] and dialectical behavior therapy (DBT) [13]. Bramham et al. reported on a group intervention for adults with ADHD resulted in greater improvement on measures of knowledge about ADHD, self-efficacy and self-esteem than the control group [14]. Rostain et al. reported robust effects for individual therapy combined with medication [15].

All of these treatments were brief, structured, skills based and included a component where the patient practiced learned skills in real life situations. While the effect size on ADHD was large in all studies, depression, anxiety, anger, and self esteem did not always improve. The early pilot studies had various methodological limitations including small sample size, with open without blinded raters, and/or did not control for comorbidity or medication.

There are now four manuals for psychological treatment of adults with ADHD, which enable replication and use of these interventions by clinicians [16-19]. The last year has seen the publication of three randomized controlled trials of CBT that are methodologically rigorous in diagnosis, use of objective outcomes, blinded raters and comparison with a non-specific form of treatment [17,20,21]. Solanto did not find that stratification by medication status changed her findings [21]. These studies demonstrated definitively that both individual and group administered CBT treatment for adults with ADHD is highly effective.

At the present time a seven site study in Germany is specifically addressing the issue of the relative benefits of medication alone, psychological treatment alone or combination therapy [22]. The therapy used in Philipsen's study is an extension of the pilot work of Hesslinger using DBT.

It remains unclear from previous studies which included either medicated or a mixed sample of medicated and unmedicated patients, whether therapy vs. medication target different outcomes., There is some evidence that therapy adds significantly to the functional and/or symptom outcome of medication alone. Wilens found that the addition of CBT improved outcomes in patients who had a partial response to medication [10]. Safren has examined whether the addition of therapy augments the improvement obtained with medication alone [8,9] and found that combination therapy was superior to medication alone. Emilsson found that CBT and medication was superior to treatment as usual and medication [20].

None of these research studies ask the opposite question: does medication add significantly to the functional and/or symptom outcome of therapy alone? To answer this question it is necessary to compare the outcome of patients who receive therapy and medication to patients who receive therapy and placebo, since there is going to be a strong halo effect of knowing you are on a medication to treat ADHD.

All of the trials cited above demonstrated that CBT is effective, and can augment the effect of medication alone. However, none of these studies controls for the effect of medication on therapy outcome. We do not know whether symptom remission with medication is a prerequisite for the ability to acquire skills in therapy. Nor do we know if medication and therapy target symptoms and functioning respectively, or if both modalities have a comparable effect on either outcome. The objective of the study reported here is to determine whether CBT psychotherapy in combination with medication is superior to CBT psychotherapy and placebo in mitigating core symptoms of ADHD and functioning.

## Methods

### Design

This was a five site randomized, placebo-controlled, parallel-group study conducted in the United States and Canada. The method of the primary outcome study is previously described in detail [23]. The primary outcome was a randomized placebo controlled study of the efficacy of stimulant, antidepressant, or both in combination with CBT. ADHD symptoms responded to stimulant, and mood/anxiety symptoms responded to antidepressant. A lifetime history of a mood disorder significantly attenuated the likelihood of response to stimulant.

The design of the secondary analyses reported here was planned a priori in the study protocol. Patients randomized to psychological treatment with stimulant (CBT/DEX) were compared to patients randomized to psychological treatment and placebo (CBT/PLB) to determine if the addition of stimulant led to a more robust response in ADHD. Both patients and therapists were blind to treatment assignment. Patients were block randomized in groups of four by a pharmacist independent of the study protocol prior to obtaining baseline measures using a computer generated randomization program. Block randomization was used to assure that there would be a sufficient sample within each group at the five sites.

### Study setting

Participants were recruited from the patient pool in the ADHD clinics at the Montreal Children's Hospital, Children's and Women's Health Centre in British Columbia, Yale University, Centre for Addictions and Mental Health, Toronto, and Duke University Medical Centre. The study data was collected between 2000 and 2002.

### Participants

**Figure 1:**
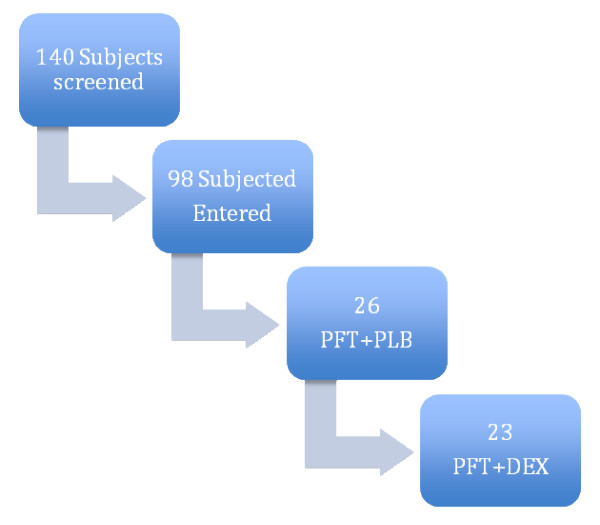
**Patient Flow**.

All participants were between 18 and 66 years of age with a primary diagnosis of ADHD confirmed by the Conners' ADHD Diagnostic Interview [24]. Patients with severe comorbidities that required treatment in their own right such as eating disorders, substance abuse, organic neurological conditions, psychosis, or suicidal ideation were excluded. Other comorbid conditions common in adults with ADHD such as learning disability, oppositional defiant disorder, depression or anxiety were included. *Comorbidity was measured with the SCID-1 as well as by clinician diagnosis in the patient's medical record*. There were no significant demographic differences between the treatment groups on age or gender. The mean age for the CBT+DEX group was 35.3 years, SD 10.4; *vs*. CBT+PLB 35.9 years, SD 9.4 (*t*(46) = .20, *p *= .84). The CBT+DEX group was 65.2% male *vs*. CBT+PLB 80% male (*Χ*^2^(1,N = 48) = 1.33, *p *= .25) (Figure 1).

Ninety-eight adults were entered into the protocol but of these only those assigned specifically to Problem Focused Therapy and Dexedrine (PFT+DEX) (N = 23) or problem focused therapy and placebo (N = 25) were entered into the protocol. Three subjects discontinued from PFT+DEX and 2 subjects discontinued from PFT+PLB. There was no significant association between study completion and the assignment to a medication treatment (62% completion) versus placebo treatment (77% completion). There was no statistically significant difference between treatment arms in discontinuation for adverse events overall. There was no significant association between study completion and the assignment to medication treatment (62% completion) versus placebo treatment (77% completion).

### Measures

Two co-primary outcomes were evaluated. Change in ADHD symptoms from baseline was measured with the investigator administered ADHD-RS-Inv, a DSM-IV symptom checklist that has been validated in children [25]. The investigator rating was based on the patient's report on the items of the ADHD-RS-Inv, but could be modified by actual observation, further questioning in an interview format asking for specific examples, information from collateral informants, and information derived from other scales such as the patient self report and collateral informant on the Conners' Adult ADHD Rating Scales [26]. Given that the focus of the psychological intervention was on functional improvement, patient functioning was measured by self report on the Sheehan Disability Scale [27], a well validated scale measuring social, family and work functioning completed by the patient [27].

### Interventions

#### Psychological treatment

All patients received individual Cognitive Behavior Therapy (CBT). The psychological treatment was developed and manualized in a series of weekly telephone conference calls with the principal investigators (Margaret Weiss, Lily Hechtman, Keith Conners, Tom Brown, and Umesh Jain) and the clinicians involved in the study. The manual documented the approach (structured, skills based, problem focused), and methods of managing possible challenges in treatment and provided modules for addressing specific issues such as emotional dysregulation, sleep, addiction, anger outbursts and other problems common in ADHD.

**Figure 2:**
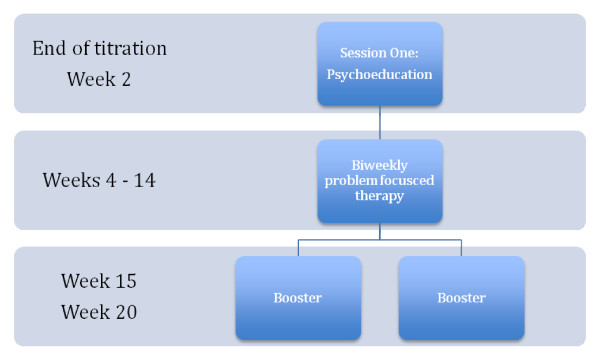
**Treatment Schedule**.

The therapy was administered individually for nine sessions. The first session took place following the completion of titration of medication when the patient was on a stable dose. This first session provided psychoeducation explaining ADHD as a neurobiological disorder and helping the patient understand the relationship between symptoms, his or her life story, and current functional impairments. Patients were seen in acute treatment every 2 weeks (for 7 sessions) and then twice in follow-up booster sessions at weeks 15 and 20 (Figure 2).

The Problem Focused Therapy was structured so that the patient chose one critical problem in their lives, such as finishing school, fighting with their boss, waking up in the morning, or conflict in family relations. Whatever the life problem they selected it was used as a vehicle for teaching the basic tools required for improved functioning. This include cognitive behavior therapy, but it also included executive functioning skills, problem solving, prioritizing, learning to self reward, delegating and setting up their environment to optimize their strengths, working to identify methods of avoiding distraction and disinhibition, and so on.

The therapy is not unlike the manual developed by Steve Safren in that the patient selected a particular goal. While this might vary from patient to patient, in all cases the limiting skills necessary to meet the goal were comparable. For example, one patient selected "being able to be consistent and to relax" as his goal. To acquire this objective it was pointed out that he would need to structure his work time efficiently through a to do list and an agenda as well as a time for regular exercise and relaxation and follow this schedule consistently. The skills developed in the process of working on this particular problem included use of a computer planner, identifying leisure as a priority, increasing efficiency at work so as to free up time, identification of cognitive distortions ("I do not deserve to exercise if I haven't got anything done") and simple interventions such as assisting him in following through in registering with a gym. As new skills emerge they are identified as such to assist the patient in generalizing these new coping strategies to other problem situations.

The therapy manual described the psychoeducation session, the approach of the therapy, common problems experienced in therapy with patients with ADHD, and approaches to the most common problems selected by patients. Specific modules were developed to be referenced by the therapy as appropriate for the problem the patient described. The modules included: anger management, work functioning, money management, self care (sleep, nutrition, exercise, driving, substance use), parenting, organization, time management, stress management, school, procrastination, marital conflict, emotional lability, self esteem, money management, and developmental transitions and role functioning. The manual is available from the authors upon request.

The format of each session included review of implementation of skills from the past week, a review of symptoms, discussion of success or difficulty with implementation of the skills already covered, and introduction of new skills for the week to follow. After much discussion the manual did not use the term "homework" to describe practice of skills between sessions, out of concern that many ADHD patients would have a negative association with the term. Each session ended with a plan for how the patient would carry through skills in the week ahead, and each session began by looking at the successes and failures to follow through.

The last two sessions (Sessions 8 and 9) were called boosters and were administered at monthly at week 15 and week 20. These "booster sessions" were meant to assist the patient in maintaining the changes that had been established, and to deal with issues of termination and follow up. The booster sessions highlighted for the patient the specific skills that had been acquired in dealing with the problem they had chosen and ways in which the same skill set could also be applied to other areas of impairment in the patient's life.

The therapy employed the key principles of CBT in challenging cognitive distortions such as personalization, overgeneralization, selective attention, disqualifying benefits, jumping to conclusions, should statements and catastrophizing-all of which are common in ADHD adults [28]. Although the therapy was based on CBT principles as applied to a real life problem, and although the therapy was both manualized and sessions were taped, we did permit therapists to be flexible and draw on other types of psychological intervention such as behaviour therapy, interpersonal therapy, environmental restructuring, or cognitive remediation if the therapist felt they would be useful or appropriate in addressing the patients chosen problem focus.

The therapy manual stipulated several points of method that were used consistently in all cases. The therapy was brief, highly structured, practical, skills based, and required practice in the patient's daily life. Wherever possible or appropriate the therapy provided instruction on the relationship between ADHD and dysfunctional symptoms. With the consent of patients, sessions were taped and listened to by other therapists (investigators) to assure some reliability in following the manual, but no ratings of the sessions were conducted. When ADHD behaviors themselves interfered with the progress of treatment (coming late, arguing, failure to practice, not listening), these behaviors were identified as part of the ADHD or oppositional diagnosis and addressed directly.

#### Medication treatment

Medication was encapsulated so that patients could not distinguish active medication from placebo. Stimulant medication was Dextroamphetamine dosed twice daily. A double dummy design was used in which placebo was also dosed twice daily. Medication was titrated by weekly increments to optimal dose over a four week period, with the investigator and patient blind to treatment. Optimal dose was defined as a rating on the Clinical Global Impression- Improvement scale of much or very much improved. Dextroamphetamine was initiated at 5 mg p.o. b.i.d. and increased to a maximum of 20 mg p.o. b.i.d.

Compliance was monitored and required for both psychotherapy and medication such that patients had to attend a minimum of 8 of the 9 sessions and take 80% of medication in order to remain in the protocol. Reminder calls, and various other forms of assistance were used to enhance compliance. Medication adherence was measured by pill counts on the study bottles which were returned by the patient at each visit.

### Procedures

Patients received a comprehensive evaluation at baseline, 15, and 20 weeks in order to determine if there was a lag effect differentiating psychological and medication treatment. The study was approved by the hospital review board and carried out consistent with the Declaration of Helsinki. All patients completed consent and/or assent as appropriate.

#### Statistical analyses

For each of the dependent variables (ADHD RS-Inv and Sheehan Disability Scale), a two-way repeated measures analyses of variance (ANOVA) was conducted with the within-participants factor being Time (Baseline, Week 15 and Week 20) and the between-participants variable being CBT and placebo, CBT and Dectroamphetamine. This ANOVA is an analyses which includes multiple components. First, it tests to determine if there is a difference between the treatments overall (main effect-that is, collapsing/averaging the data for each of the three time points per treatment group and seeing if one group was different from the other). Second, the ANOVA tests if there is a difference across the three repeated measures at baseline, Week 15 and Week 20 (collapsing the treatment group data to see if overall, there are differences across time for all subjects). Lastly, the ANOVA determines if there is an interaction between time and treatment-does group improve over time more than the other group.

Analyses of both groups as a whole for efficacy of CBT will be done, if it was found that there are no between group differences. All analyses were intent to treat, with last observation carried forward. Given the total sample size of 48, an alpha of .5, and an effect size of 1.1 we had a greater than 90% chance of finding a difference between the groups if there had been one.

## Results

Three participants discontinued before the trial ended in the Dextroamphetamine group and two participants in the placebo group. Adverse events have been reported in detail elsewhere [23]. All adverse events were mild to moderate and there was no statistically significant difference between the groups.

The means and standard deviations for the ADHD RS-Inv are presented in Table 1.

**Table 1:** ADHD RS-Inv

Time	Treatment	Mean	SD	N	*p*
Baseline	CBT+DEX	33.35	8.21	23	.77
	
	CBT+PLB	32.68	7.62	25	

Week 15	CBT+DEX	20.87	9.32	23	.21
	
	CBT+PLB	24.96	12.25	25	

Week 20	CBT+DEX	20.78	9.65	23	.39
	
	CBT+PLB	23.56	12.39	25	

Outcome in ADHD is often measured in three ways: improvement, response and full remission. We elected to look at the two extremes: patients who showed a significant change with treatment even if they did not have a full response, and patients who achieved normalization. We defined improvement conservatively as a 25% drop in the ADHD-RS-Inv score and remission as a mean score less than or equal to 1 [29]. Using these definitions 63% of patients improved, and 47% percent of patients achieved remission.

**Figure 3:**
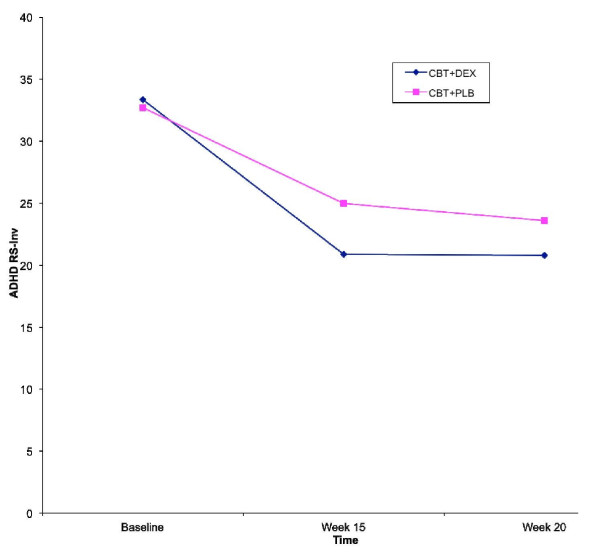
**ADHD RS-Inv by Time and Treatment**.

Table 1 and Figure 3 show the mean values for each of the treatment conditions across the three assessment times, and shows improvement in ADHD RS-Inv values across time in both treatment conditions. Independent samples *t *tests were conducted comparing the treatments at each point of time. There were no statistical differences between groups at baseline or at any other time in follow up. There was an absolute difference favoring CBT+DEX vs. CBT+PLB at each point in treatment, that did not reach statistical significance. At week 15 the change score for CBT+DEX was 12.5 *vs*. 7.9 for CBT+PLB. This pattern continued such that at week 20 the decrease in ADHD symptoms for CBT+DEX was 12.6 *vs*. CBT+PLB 9.2. There was not a significant interaction between Time and Treatment, *F*(2, 46) = 2.21, *p *= .12. Testing of the between-participants effect of Treatment did not show a significant main effect, *F*(1,46) = 0.63, *p *= .43.

Although there was no difference between CBT+DEX and CBT+PLB, both groups showed robust improvement over time. Tests of within-participants effects showed a significant effect of Time, *F*(2,) = 53.72, *p *< .001. Given that medication vs. placebo had no differential impact on outcome, we examined the effect size of improvement for the sample as a whole to determine the impact of CBT. The effect size (Cohen's d using the pooled SD) based on improvement obtained at 15 weeks was 1.1. This was maintained at 20 weeks where the effect size of improvement was unchanged at 1.1.

Results for the Sheehan Disability Scale have been reported in Table 2.

**Table 2:** Sheehan Disability Scale

Time	Treatment	Mean	SD	N*	*p*
Baseline	CBT+DEX	18.8182	7.39	22	.29
	
	CBT+PLB	20.8800	5.87	25	

Week 15	CBT+DEX	14.7727	8.65	22	.31
	
	CBT+PLB	17.3600	8.19	25	

Week 20	CBT+DEX	14.6364	9.07	22	.15
	
	CBT+PLB	18.2400	7.78	25	

*One subject in CBT/DEX did not provide data at week 15 and was excluded from the analyses

**Figure 4:**
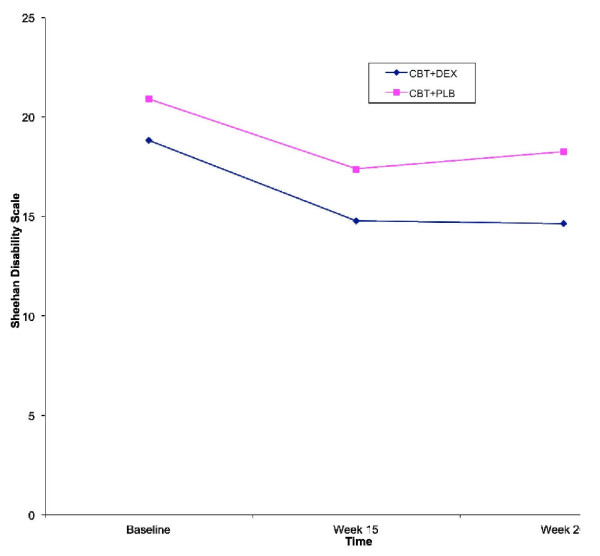
**Sheehan Disability Scale by Time and Treatment**.

Mean Sheehan Disability Scale values for each of the treatment conditions across the three assessment times are shown in Table 2 and Figure 4. The CBT+DEX group shows improvement in functioning by Week 15 and this improvement is sustained at Week 20. The CBT+PLB group also shows improvement in functioning at Week 15, but this improvement is attenuated somewhat at Week 20. Tests of within-participants effects showed a significant effect of Time, *F*(1, 46) = 12.70, *p *< .001, but no significant interaction between Time and Treatment, *F*(1, 45) = 0.45, *p *= .64. There was not a significant main effect of Treatment on the Sheehan Disability Scale, *F*(1,45) = 1.73, *p *= .20. Given no significant difference between medication and placebo we calculated the effect size of improvement in functioning for all participants at 15 weeks which was .5, and .44 at 20 weeks.

## Discussion

This is the first report of a double blind, randomized controlled study comparing the efficacy of CBT therapy with either stimulant or placebo for adults with ADHD followed over five months. All patients showed significant improvement, going into remission and maintaining those gains over the 20 weeks. This supports the work of Solanto and Safren [17,21] which showed that adults may benefit from short term, structured CBT therapy. Furthermore, both therapy and medication treatment effects were enduring, even when therapy sessions were being tapered. The effect size of treatment on symptoms was large and the effect on actual functioning was moderate.

Although the power analyses demonstrates that we were adequately powered to show a difference if medication impacted outcome, it is impossible to prove the null hypothesis. It can be stated that if there is such an effect it would take a large sample size to demonstrate it, and its clinical relevance to the majority of patients would be modest. Nonetheless, we recognize it is never possible to prove the null hypothesis, and that absence of evidence is not evidence of absence. A limitation of our design is it is impossible to distinguish the extent to which the outcome of placebo/stimulant and CBT represents a specific effect of either intervention. We did not control for site to site differences. Therapy was administered by more than ten therapists, and although all the therapists participated in development of the manual and sessions were taped and listened to by therapists across sites, there was no attempt made to guarantee treatment fidelity. This limits the capacity to replicate the findings of this study. The findings are likely to be generalizable in that the population came from five very different geographic regions, and the range of functioning of the patients was highly variable and consistent with the population seen in ADHD. The study cannot tell us the extent to which medication or therapy effects endured beyond the five months of the study. The study failed to demonstrate that medication and therapy was statistically superior to therapy alone, but failure to find a difference is not akin to demonstrating equivalence.

## Conclusions

This study supports previous findings that adults with ADHD are responsive to CBT intervention. It extends previous research in demonstrating that the impact of CBT can be demonstrated in both response of core symptoms and functioning, whether administered with stimulants or placebo. This is the first randomized, placebo controlled study to demonstrate that where CBT augments the effect of medication, we were not able to demonstrate that medication impacts on the capacity to learn the skills taught in CBT. This suggests that CBT therapy can be effective in adults with ADHD, even in patients who are not able to use stimulants.

Unlike findings on combination therapy vs. medication alone in children CBT [30], this study suggests that CBT is an effective treatment for adults with ADHD with or without stimulant therapy. Future research is needed to replicate the power and methodology of the Multimodal Treatment of ADHD study in children in an adult population. It is possible that when children are medicated they are in learning environments where they can acquire the attention skills they need. By contrast, adults who have had ADHD as children and continue to have ADHD, may be quite different to children in that the key issue is no longer simply remission of symptoms, but providing them with the opportunity to aquire the attention skills they failed to develop in childhood.

## Competing interests

This project was funded by GlaxoSmithKline. Margaret Weiss and Lily Hechtman have received honoraria, speaking fees, consultation fees and research funds from Eli Lilly, Purdue, Janssen, and Shire but do not hold stocks in any pharmaceutical company. There are no other competing interests by these or the other authors.

## Authors' contributions

MW was the lead author and conceptualized the design of the analyses. MW designed and carried out the statistical analyses. BG helped design and carry through the CBT treatment and edited each version of the manuscript to reflect its clinical relevance. LG coordinated the research, and participated in writing the paper to assure it was consistent with the CONSORT design. LH designed the research, and participated in every aspect of the study from its inception and the production of this manuscript. All authors read and approved the final manuscript.

## Pre-publication history

The pre-publication history for this paper can be accessed here:

http://www.biomedcentral.com/1471-244X/12/30/prepub
